# An Essay on Intestinal Auscultation

**Published:** 1850-01

**Authors:** 


*An Essay on Intestinal Auscultation.* By CHARLES HOOKER, M. D. Professor of Anatomy and Physiology in Yale Medical College.

This is a well written pamphlet of about ten pages, read at the annual meeting of the Connecticut Medical Society in April, 1847, and reprinted from the Boston Medical and Surgical Journal. In it is explained the application of auscultation to the sounds produced in the intestinal canal by the movements of it's contents.

"These sounds," says the author, "are varied by many causes, such as the quickness, regularity, and other variations of the peristaltic action, the degree of fulness of the intestines, the proportions of the gaseous and other contents, the fluidity of the liquid contents, &c. The sounds thus varying with the causes of their production, afford indication of these several causes; and they thus become signs of actions and conditions of the intestines, a knowledge of which is of the utmost importance in investigating the diseases of these viscera. In most diseases of the intestinal canal the sounds do not afford definite diagnostic signs to characterize the different diseases, like the diagnostic signs disclosed by auscultation in thoracic diseases. They are chiefly signs of particular conditions or actions, which may occur in various intestinal diseases, rather than diagnostic signs to distinguish different diseases. In some diseases, however, signs are thus obtained, which perhaps may be considered as truly diagnostic of the diseases in which they occur.

In the *Asiatic Cholera*, which prevailed in New Haven in 1832, this application of auscultation was attended with interesting results, which were noticed in an account of the cases which came under my observation, published in the Boston Medical and Surgical Journal for July 1833. Writers generally noticed the loud borborygmi, audible at a distance from the patient, which occurred in that disease; and to the ear applied over the abdomen the sounds were so peculiar—at least so different from what I have observed in other diseases—that they seemed distinctly characteristic of that disease. These sounds manifested a rapid commotion of the whole intestinal canal, and might be compared to those produced by shaking together several flasks of various sizes partly filled with water. Frequently the sounds appeared to indicate that the rapid peristaltic motions were suddenly arrested and reversed by an anti-peristaltic action, which occurrence immediately preceded a paroxysm of vomiting. The large quantity of serum effused into the intestines, causing an extreme fluidity of their contents with the rapid and irregular peristaltic and anti-peristaltic motions, would sufficiently account for this unusual variety of sounds.

The effects of various remedies upon the intestinal action, as indicated by the sounds, were carefully observed. Practitioners were generally disappointed, in that disease, to find the frequent vomiting and purging not checked by the administration of stimulants and astringents; and the sounds manifestly indicated that the common effect of these remedies was decidedly to increase the intestinal commotion. Such was the manifest effect of opium, unless given in doses so large as to produce alarming prostration. On the contrary, frequent small doses of camphor, with a free administration of ice, appeared to have a soothing operation in moderating the rapid and irregular intestinal action. The comparative effects of large and small doses of calomel were strikingly interesting. Frequent small doses did not seem to diminish, but, at least temporarily, to increase the disordered peristaltic and anti-peristaltic motions; while a single drachm dose almost invariably caused a total suspension of these motions. Calomel, in very large doses, thus seemed to be the appropriate remedy for the disease. It appeared to overpower the diseased intestinal action, arrested the vomiting and purging, and caused a total suspension of all intestinal motion, during which no sound was audible. An interval of perfect intestinal silence and repose now continued, ordinarily from eight to twelve hours, after which a natural peristaltic murmur indicated a gradual return of healthy action, which was in time succeeded by the grass-green evacuations, commonly regarded as evidence of a favorable crisis of the disease. Thus the large doses of calomel, instead of exhausting the system by an excessive cathartic operation, actually obviated exhaustion by arresting the profuse serous evacuations attending the disease.

Ordinarily the danger was considered as overcome, when the disordered intestinal action was suspended, and the stage of repose produced; and in this town few cases terminated fatally, when the practice was adopted of effecting this result by the large doses of calomel, before the system had been extremely exhausted by evacuations."

The application of auscultation to the diagnosis of other intestinal affections is exceedingly interesting, and worthy the study of practitioners; for instance, the author states, that in ordinary colic there is an *incipient*, *forming*, or *latent* stage, in which, although no acute pain be present, there are symptoms such as precede the cold stage of an intermittent fever; languor, lassitude, &c. The abdominal sensations, although not often noticed at the time, are often described as a cold, heavy, numb feeling between the region of the stomach and umbilicus.

In this stage a perfect stillness is discovered within the abdominal cavity. Occasionally there is a slight rumbling in the course of the large intestine, but no indication of movement in the small bowel. This stage is deserving of particular attention, because in it, the peristaltic action is readily restored and the violent symptoms averted by slight friction, a hot draught, or some aromatic, or antispasmodic, as a few grains of camphor.

If the patient be examined, however, during the violent symptoms, the ear will discover *no evidence of peristaltic motion*; and this the author asserts to be a chief " *essential character of colic*" occasionally, however, slight accidental movements take place from abdominal pressure; or, anti-peristaltic motions occur producing either nausea or vomiting, these are only occasional, however, and transient. As the cessation of peristaltic action is an essential character of colic, so is the return of this movement, extending throughout the intestinal tube, indicative of a favorable crisis; " when this description of sounds is heard," says our author, " the patient may be considered as safe, even if the pain continues severe." A subsidence of pain, without the return of peristaltic action, affords no favorable indication, indeed is significative of great danger, if the strength be exausted by extreme suffering.

Usually, the return of movement is accompanied by a cessation of pain; but in protracted cases, where inflammation has supervened, peristaltic action may be accompanied by pain. This is similar to what is often observed during the resolution of pneumonia, when a return of respiration to the inflamed lung, previously impermeable, produces the keenest pain. " In such cases, auscultation informs us that all is well, even when the sensations of the patient would indicate an aggravation of the disease. The signs, above mentioned, in colic, will often indicate that further medication is unnecessary, even before the general symptoms show any sign of mitigation, and frequently enable the practitioner, under the same circumstances, to give a cheering prognosis.

The author also explains how in lead colic, by the same means, much valuable information may be gained as to its progress and duration. So again in dysentery.

In the management of cathartic medicines much discretion is often needed, as to their administration or withdrawal. By the application of the ear, it may be discovered that spontaneous evacuations are about to occur. Or when cathartics have been administered, knowledge of their speedy operation is obtained, and the ill effects of over dosing obviated.

In diarrhœa, too, this method of exploration is valuable, says the author, as affording an estimate of the severity and obstinacy of the disease, or an immediate assurance from the cessation of abdominal sounds that there is no danger.

We wish that space were allowed us to give a full analysis of this excellent paper. Enough, however, we trust, has been given to prove its usefulness, and to stimulate others to the employment of this valuable diagnostic means, as well as to show the wide range of its applicability.

